# An algorithm for judging and generating multivariate quadratic quasigroups over Galois fields

**DOI:** 10.1186/s40064-016-3525-2

**Published:** 2016-10-22

**Authors:** Ying Zhang, Huisheng Zhang

**Affiliations:** Department of Mathematics, Dalian Maritime University, Dalian, 116024 China

**Keywords:** Quasigroup, Multivariate quadratic quasigroup, Vector-valued boolean functions, Judging method, Generating algorithm

## Abstract

As the basic cryptographic structure for multivariate quadratic quasigroup (MQQ) scheme, MQQ has been one of the latest tools in designing MQ cryptosystem. There have been several construction methods for MQQs in the literature, however, the algorithm for judging whether quasigroups of any order are MQQs over Galois fields is still lacking. To this end, the objective of this paper is to establish a necessary and sufficient condition for a given quasigroup of order *p* ^*kd*^ to be an MQQ over $GF(p^{k})$. Based on this condition, we then propose an algorithm to justify whether or not a given quasigroup in the form of multiplication table of any order *p* ^*kd*^ is an MQQ over $GF(p^{k})$, and generate the *d* Boolean functions of the MQQ if the quasigroup is an MQQ. As a result, we can obtain all the MQQs over $GF(p^{k})$ in theory using the proposed algorithm. Two examples are provided to illustrate the validity of our method.

## Background

With the development of quantum computer, post-quantum cryptography (PQC) has gained intensive attention in recent years. Multivariate Public Key Cryptography (MPKC) is one among a few serious candidates to have risen to prominence as post-quantum options. In the last two decades, MPKC was developed rapidly, with many schemes being proposed, attacked and then amended. Based on multivariate quadratic quasigroups (MQQ), Gligoroski et al. recently proposed a novel type of MPKC-MQQ schemes (including both the signature scheme and the encryption scheme) (Gligoroski et al. 2008, 2011). As these schemes only need the basic operations of XOR and AND between bits during the encryption and decryption processes, they attain the speed of decryption/signature generation comparable to a typical symmetric block cipher (Hadedy et al. 2008). The size of the set of MQQs is rather large, which makes MQQ scheme have a bigger scale of private key and public key than conventional MPKC schemes (Gligoroski et al. 2008). Moreover, these schemes offer flexibility in their implementation from parallelization point of view (Hadedy et al. 2008). In a recent work, MQQ schemes have been successfully used in wireless sensor network (Maia et al. 2010).

As the basic step for the MQQ scheme, generating MQQ is an important and challenging task. Gligoroski et al. established a sufficient condition of generating an MQQ for a given quasigroup (Gligoroski et al. 2008). Based upon this condition, a randomized generation algorithm for MQQs was also proposed therein. However, this algorithm is time-consuming and can only generate MQQs of order $2^d(d\le 5)$. Subsequently, an improved algorithm to generate MQQs was proposed by Ahlawat et al. (2009), and the existence of MQQs from *d* = 2 to *d* = 14 was verified. Recently, the sufficient condition in Gligoroski et al. (2008) was simplified by Chen et al. (2010) and an efficient algorithm for generating bilinear MQQs (a subclass of MQQs) of any order 2^*d*^ was proposed. In addition, new algorithms and theory for generating MQQs are also reported by Samardjiska et al. (2010) and Christov (2009), respectively.

Different from the aforementioned work on constructing MQQs, equipped with a new necessary and sufficient condition for bilinear MQQ, Zhang and Zhang (2013) proposed an algorithm for judging and generating bilinear MQQ from the multiplication table of a quasigroup, thus answering the question how to judge whether or not an arbitrary quasigroup is a bilinear MQQ and providing a feasible way to generate all the bilinear MQQs in theory. Considering that bilinear MQQs are only a subclass of MQQs and the algebraic operation of Zhang and Zhang (2013) is only limited to *GF*(2), the objective of this paper is to extend the previous work (Zhang and Zhang 2013) by bringing out a solution on how to judge and generate MQQ over Galois fields. Specifically, we make the following contributions:

1. We establish a necessary and sufficient condition for a quasigroup of any order *p* ^*kd*^ to be MQQ over $GF(p^{k})$, which answers a theoretical question: when is a quasigroup an MQQ over $GF(p^{k})$?
2. Based on the above condition, we propose an algorithm for justifying whether or not a given quasigroup of order *p* ^*kd*^ is an MQQ over $GF(p^{k})$ and generating all its boolean functions if the quasigroup is an MQQ.
3. Compared with the previous work (Zhang and Zhang 2013), the strategy proposed in this paper can identify all the MQQs, including both bilinear MQQs and non-bilinear ones. Moreover, the algebraic operation in Galois fields provides more flexibility in choosing *p*, *k* and *d*, which is useful for applying MQQ-design to various platforms and also benefits us to find more MQQs.

The remainder of the paper is organized as follows. Second section recalls the original MQQ generation scheme (Gligoroski et al. 2008). Third section proposes a necessary and sufficient condition and an algorithm for justifying and generating MQQs in $GF(p^{kd})$. Two examples are provided to show the validity our algorithms in fourth section. Finally, we conclude the paper in last section.

## Original MQQ generation scheme

### **Definition 1**

[*Definition 1 in* Chen et al. (2010)] A quasigroup $(Q,*)$ is a set *Q* with a binary operation $*$ such that for any $a,b\in Q$, there exist unique *x*, *y*:

1 $$x*a=b;\quad a*y=b.$$

### **Lemma 1**

[Lemma 1 in Gligoroski et al. (2008)] *For every quasigroup* $(Q,*)$ *of order* $2^d$ *and for each bijection* $Q\rightarrow \{0,1,\ldots ,2^d-1\}$ *, there are a uniquely determined vector valued Boolean function* $*vv$ *and d uniquely determined* 2*d* *-ary Boolean functions* $f_1,f_2,\ldots ,f_d$ *such that for each* $a,b,c\in Q$

2 $$\begin{aligned}a*b&=c \Longleftrightarrow *vv(x_1,\ldots ,x_d,x_{d+1},\ldots ,x_{2d}) \\&=(f_1(x_1,\ldots ,x_d,x_{d+1},\ldots ,x_{2d}),\ldots ,f_d(x_1,\ldots ,x_d,x_{d+1},\ldots ,x_{2d})). \end{aligned}$$

In general, for a randomly generated quasigroup of order $2^d (d\ge 4)$, the degrees of Boolean functions are usually higher than 2. Such quasigroups are not suitable for the construction of multivariate quadratic public-key cryptosystem.

### **Definition 2**

[*Definition 3 in* Gligoroski et al. (2008)] A quasigroup $(Q,*)$ of order 2^*d*^ is called multivariate quadratic quasigroup (MQQ) of type $Quad_{d-k}Lin_k$ if exactly $d-k$ of the polynomials $f_s$ are of degree 2 and *k* of them are of degree 1, where $0\le k<d$.

## Main results

In this section, we first establish a necessary and sufficient condition for a given quasigroup of order *p* ^*kd*^ to be an MQQ over $GF(p^{k})$, and then use this condition to propose an algorithm for justifying whether or not a quasigroup of order *p* ^*kd*^ is an MQQ over $GF(p^{k})$ and generating *d* Boolean functions of MQQ if it is.

For convenience the following notations are adopted: $I_n$ denotes the identity matrix of order *n*; $E_{i,j}$ is the shorthand for the elementary matrix of switching all matrix elements on row *i* with their counterparts on row *j* of $I_n$; $E_{i,j}(1)$ denotes the elementary matrix of adding all matrix elements on row *j* (column *i*) to their counterparts on row *i* (column *j*) of $I_n$.

### Necessary and sufficient condition for MQQs over $GF(p^{kd})$

#### **Definition 3**

[*see* Golub and Loan (1996)] Given an ${m\times n}$ matrix $A=(a_{ij})$, $\overline{vec}(A)$ is a vector defined as

$$\overline{vec}(A)=(a_{11},\ldots ,a_{1n},a_{21},\ldots ,a_{2n},\ldots ,a_{m1},\ldots ,a_{mn})^T.$$

#### **Lemma 2**

[see Golub and Loan (1996)] *Let* $A\in R^{m\times u},B\in R^{v\times n}, X\in R^{u\times v}$ *, then*

$$\overline{vec}(AXB)=(A\otimes B^T)\overline{vec}(X).$$

#### **Lemma 3**

*Let* $A=(a_{ij})_{m\times u},B=(b_{lt})_{v\times n}, X=(x_{jl})_{u\times v}$, where $a_{ij},b_{lt},x_{jl}\in \{0,1,\ldots ,p^k-1\},$ *and p be prime number, then*

$$\overline{vec}(AXB\mod p^k)=(A \otimes B^T \mod p^k)~\overline{vec}(X)\mod p^k.$$

#### **Lemma 4**

[see Golub and Loan (1996)] *Let A, B, C, D be suitably sized matrices. Then*

$$(A+B)\otimes (C+D)=A\otimes C+A\otimes D+B\otimes C+B\otimes D.$$

Let a quasigroup $(Q,*)$ of order *p* ^*kd*^ be given by the multiplication scheme in Table 1, where $q^{(j)}_i\in Q$, $(i,j=0,1,\ldots ,p^{kd}-1)$. For given *i* and $\forall j\ne j^\prime$, we have $q^{(j)}_i\ne q^{(j^\prime )}_{i}$; for given *j* and $\forall i\ne i^\prime$, we have $q^{(j)}_i\ne q^{(j)}_{i^\prime }$. One can choose two bijections $\kappa : Q\rightarrow \{0 , 1,\ldots ,p-1\}^{dk}$ and $\iota :\{0,1,\ldots ,p-1\}^k\rightarrow \{0,1,\ldots ,p^k-1\}$. Collect the elements of Table 1 into a vector

3 $$\left( q^{(0)}_0, q^{(0)}_1, \ldots , q^{(0)}_{p^{kd}-1}, q^{(1)}_0, q^{(1)}_1, \ldots , q^{(1)}_{p^{kd}-1}, \ldots , q^{(p^{kd}-1)}_0, q^{(p^{kd}-1)}_1, \ldots , q^{(p^{kd}-1)}_{p^{kd}-1} \right) ^T,$$

and convert every element of the vector into a *kd*-ary sequence over *GF*(*p*) according to the bijection $\kappa$. Then, divide every *kd*-ary binary sequence into *d* groups from left to right, where every group is a *k*-ary sequence, and represent every group by a unique element in $\{0,1,\ldots ,p^k-1\}$ according to the bijection $\iota$. In this way, we obtain a $p^{2kd}\times d$ matrix $[b_1,\ldots ,b_d]$, where every $b_s(s=1,\ldots , d)$ is a $p^{2kd}$ dimensional column vector over finite field $GF(p^k)$

**Table 1:** A quasigroup $(Q,*)$ of order *p* ^*kd*^

*	0	1	2	$\ldots$	$p^{kd}-1$
0	$q^{(0)}_0$	$q^{(0)}_1$	$q^{(0)}_2$	$\cdots$	$q^{(0)}_{p^{kd}-1}$
1	$q^{(1)}_0$	$q^{(1)}_1$	$q^{(1)}_2$	$\cdots$	$q^{(1)}_{p^{kd}-1}$
2	$q^{(2)}_0$	$q^{(2)}_1$	$q^{(2)}_2$	$\cdots$	$q^{(2)}_{p^{kd}-1}$
$\vdots$	$\vdots$	$\vdots$	$\vdots$	$\vdots$	$\vdots$
$p^{kd}-1$	$q^{(p^{kd}-1)}_0$	$q^{(p^{kd}-1)}_1$	$q^{(p^{kd}-1)}_2$	$\cdots$	$q^{(p^{kd}-1)}_{p^{kd}-1}$

.

According to Lemma 1, whether a given quasigroup is an MQQ over $GF(p^k)$ mainly lies in whether there is 2*d*-ary quadratic Boolean function set $\{f_1,f_2,\ldots ,f_d\}$ satisfying Table 1. Note that, any $f_s(x_1,\ldots ,x_d,x_{d+1},\ldots ,x_{2d})$ can be written in the form

4 $$f_s=(1,x_1,\ldots ,x_d,x_{d+1},\ldots ,x_{2d}){\mathcal {A}}_s\left( \begin{array}{c}1\\ x_1\\ \vdots \\ x_d\\ x_{d+1}\\ \vdots \\ x_{2d}\end{array}\right) ,\quad (s=1,2,\ldots , d) ,$$

where ${\mathcal {A}}_s$ is a matrix of order ${2d+1}$ over finite field $GF(p^k)$. By (2) and Table 1, when $(x_1,\ldots ,x_d)$ and $(x_{d+1},\ldots ,x_{2d})$ are respectively assigned *d*-ary sequences in the order of $\{0,1,\ldots ,p^{kd}-1\}$ in which every element is written by *d*-ary sequence over $GF(p^k)$, namely, $(1,x_1,\ldots ,x_d,x_{d+1},\ldots ,x_{2d})$ in $f_s$ are assigned all row vectors of the following ${p^{2kd}\times (2d+1)}$ matrix of the form

5 $$\left( \begin{array}{ccccccccc} 1 &{} 0 &{} \cdots &{} 0 &{} 0 &{} 0 &{}\cdots &{}0 &{} 0\\ \vdots &{} \vdots &{} \vdots &{} \vdots &{}\vdots &{} \vdots &{} \vdots &{} \vdots &{}\vdots \\ 1 &{} 0 &{} \cdots &{} 0 &{} 0 &{} 0&{}\cdots &{}0&{} p^k-1\\ \ddots &{}\ddots &{} \ddots &{} \ddots &{} \ddots &{} \ddots &{} \ddots &{}\ddots &{}\ddots \\ 1 &{} 0 &{}\cdots &{} 0 &{} 0&{} 0 &{}\cdots &{} p^k-1&{} 0\\ \vdots &{} \vdots &{} \vdots &{} \vdots &{}\vdots &{} \vdots &{} \vdots &{} \vdots &{}\vdots \\ 1 &{} 0 &{} \cdots &{} 0 &{} 0 &{} 0 &{}\cdots &{} p^k-1&{}p^k-1\\ \ddots &{} \ddots &{} \ddots &{} \ddots &{}\ddots &{} \ddots &{} \ddots &{} \ddots &{}\ddots \\ 1 &{} 0 &{} \cdots &{} 0 &{} p^k-1 &{} p^k-1 &{}\cdots &{} p^k-1&{}0\\ \vdots &{} \vdots &{} \vdots &{} \vdots &{}\vdots &{} \vdots &{} \vdots &{} \vdots &{}\vdots \\ 1 &{} 0 &{}\cdots &{} 0 &{} p^k-1&{}p^k-1 &{}\cdots &{} p^k-1&{}p^k-1\\ \ddots &{} \ddots &{} \ddots &{} \ddots &{}\ddots &{} \ddots &{} \ddots &{} \ddots &{}\ddots \\ 1 &{} p^k-1 &{} \cdots &{} p^k-1 &{} 0 &{} 0&{}\cdots &{}0&{} 0\\ \vdots &{} \vdots &{} \vdots &{} \vdots &{}\vdots &{} \vdots &{} \vdots &{} \vdots &{}\vdots \\ 1 &{} p^k-1 &{} \cdots &{} p^k-1 &{} 0 &{} 0&{}\cdots &{}0&{} p^k-1\\ \ddots &{}\ddots &{} \ddots &{} \ddots &{} \ddots &{} \ddots &{} \ddots &{}\ddots &{}\ddots \\ 1 &{} p^k-1 &{}\cdots &{} p^k-1 &{} 0&{} 0 &{}\cdots &{} p^k-1&{} 0\\ \vdots &{} \vdots &{} \vdots &{} \vdots &{}\vdots &{} \vdots &{} \vdots &{} \vdots &{}\vdots \\ 1 &{}p^k-1 &{} \cdots &{} p^k-1 &{} 0 &{} 0 &{}\cdots &{} p^k-1&{}p^k-1\\ \ddots &{} \ddots &{} \ddots &{} \ddots &{}\ddots &{} \ddots &{} \ddots &{} \ddots &{}\ddots \\ 1 &{} p^k-1 &{} \cdots &{} p^k-1 &{} p^k-1 &{} p^k-1 &{}\cdots &{} p^k-1&{}0\\ \vdots &{} \vdots &{} \vdots &{} \vdots &{}\vdots &{} \vdots &{} \vdots &{} \vdots &{}\vdots \\ 1 &{} p^k-1 &{}\cdots &{} p^k-1 &{} p^k-1&{}p^k-1 &{}\cdots &{} p^k-1&{}p^k-1\\ \end{array}\right) =\left( \begin{array}{c} {\mathbf q} _0\\ {\mathbf q} _1\\ {\mathbf q} _2\\ \vdots \\ {\mathbf q} _{p^{2kd}-1} \end{array}\right) ,$$

we know that every ${\mathbf q }_k(k=0,1,\ldots ,p^{2kd}-1)$ for any $b_s(s=1,\ldots ,d)$ needs to satisfy

6 $$\left( \begin{array}{c} {\mathbf q} _0 {\mathcal {A}}_s {\mathbf q}_0^T\\ {\mathbf q}_1 {\mathcal {A}}_s {\mathbf q} _1^T\\ {\mathbf q}_2 {\mathcal {A}}_s {\mathbf q}_2^T\\ \vdots \\ {\mathbf q}_{p^{2kd}-1} {\mathcal {A}}_s {\mathbf q}_{p^{2kd}-1}^T \end{array}\right) \mod p^k=b_s.$$

By Lemma 3, (6) can be reshaped as

7 $$\left( \begin{array}{c} {\mathbf q}_0 \otimes {\mathbf q}_0\\ {\mathbf q}_1 \otimes {\mathbf q} _1\\ {\mathbf q} _2 \otimes {\mathbf q} _2\\ \vdots \\ {\mathbf q} _{p^{2kd}-1} \otimes {\mathbf q} _{p^{2kd}-1} \end{array}\right) \overline{vec}(\mathcal {A}_s)\mod p^k=b_s.$$

Thus, the given quasigroup in Table 1 is an MQQ over $GF(p^k)$ iff there is a set of matrices $\{\mathcal {A}_1,\ldots ,{\mathcal {A}}_d\}$ satisfying the following matrix equation

8 $$\left( \begin{array}{c} {\mathbf q} _0 \otimes {\mathbf q} _0\\ {\mathbf q} _1 \otimes {\mathbf q} _1\\ {\mathbf q} _2 \otimes {\mathbf q} _2\\ \vdots \\ {\mathbf q} _{p^{2kd}-1} \otimes {\mathbf q} _{p^{2kd}-1} \end{array}\right) [\overline{vec}({\mathcal {A}}_1),\ldots ,\overline{vec}({\mathcal {A}}_d)]\mod p^k=[b_1,\ldots ,b_d],$$

where $[\overline{vec}({\mathcal {A}}_1),\ldots ,\overline{vec}({\mathcal {A}}_d)]$ is regarded as an unknown matrix $[x_1,\ldots ,x_d]$.

By now we have proved the following necessary and sufficient condition that a given quasigroup is an MQQ over $GF(p^k)$.

#### **Theorem 1**

*For a given quasigroup* $(Q,*)$ *of order* *p* ^*kd*^ *, convert every element of* $(Q,*)$ *into a kd-ary sequence over* *GF*(*p*) *according to the bijection* $\kappa$ *, divide every kd-ary sequence into d groups from left to right, and represent every k-ary sequence by a unique element in* $\{0,1,\ldots ,p^k-1\}$ *according to the bijection* $\iota$. *Then* $(Q,*)$ *is an MQQ over* $GF(p^k)$ *of type* $Quad_{d-k}Lin_k$ *if and only if the matrix equation* (8) *has solution. Furthermore,* $f_s\, (s=1,2,\ldots , d)$ *obtained by* (4) *are just d Boolean polynomials of the MQQ, and their degrees are not more than 2.*

### Proposed algorithm

Based on Theorem 1, now we begin to develop an algorithm for justifying whether or not a quasigroup of order *p* ^*kd*^ is an MQQ over $GF(p^{k})$ and generating *d* Boolean functions of MQQ if it is.

Write

$${\mathcal {Q}}_{k,d}=\left( \begin{array}{c} {\mathbf q} _0 \otimes {\mathbf q} _0\\ {\mathbf q} _1 \otimes {\mathbf q} _1\\ {\mathbf q} _2 \otimes {\mathbf q} _2\\ \vdots \\ {\mathbf q} _{p^{2kd}-1} \otimes {\mathbf q} _{p^{2kd}-1} \end{array}\right) \mod p^k,$$

then $[{\mathcal {Q}}_{k,d},b_1,\ldots ,b_d]$ is the augmented matrix associated with matrix equation (8).

According to Theorem 1, the existence of the solution to the matrix equation (8) depends on whether the rank of ${\mathcal {Q}}_{k,d}$ is equal to the rank of $[{\mathcal {Q}}_{k,d},b_1,\ldots ,b_d]$. Firstly, we compute the rank of ${\mathcal {Q}}_{k,d}$. Note that the coefficient matrix ${\mathcal {Q}}_{k,d}$ is fixed for all the quasigroups of order *p* ^*kd*^. Write

9 $$\left( \begin{array}{c} {\mathbf q} _0\\ {\mathbf q} _1\\ {\mathbf q} _2\\ {\mathbf q} _3\\ \vdots \\ {\mathbf q} _{p^{2kd}-1} \end{array}\right) =\left( \begin{array}{c} {\mathbf q} _0\\ {\mathbf q} _0+{\mathbf p} _1\\ \vdots \\ {\mathbf q} _0+(p^k-1){\mathbf p} _1\\ {\mathbf q} _0+{\mathbf p} _2\\ {\mathbf q} _0+{\mathbf p} _2+{\mathbf p} _1\\ \vdots \\ {\mathbf q} _0+{\mathbf p} _2+(p^k-1){\mathbf p} _1\\ \vdots \\ {\mathbf q} _0+(p^k-1){\mathbf p} _2\\ {\mathbf q} _0+(p^k-1){\mathbf p} _2+{\mathbf p} _1\\ \vdots \\ {\mathbf q} _0+(p^k-1){\mathbf p} _2+(p^k-1){\mathbf p} _1\\ \vdots \\ {\mathbf q} _0+(p^k-1){\mathbf p} _{2d}+(p^k-1){\mathbf p} _{2d-1}+\cdots +(p^k-1){\mathbf p} _{2}+(p^k-1){\mathbf p} _{1} \end{array}\right) ,$$

then ${\mathcal {Q}}_{k,d}$ takes the form

10 $$\left( \begin{array}{c} {\mathbf q} _0 \otimes {\mathbf q} _0\\ ({\mathbf q} _0+{\mathbf p} _1) \otimes ({\mathbf q} _0+{\mathbf p} _1)\\ \vdots \\ \left( {\mathbf q} _0+(p^k-1){\mathbf p} _1\right) \otimes \left( {\mathbf q} _0+(p^k-1){\mathbf p} _1\right) \\ ({\mathbf q} _0+{\mathbf p} _2)\otimes ({\mathbf q} _0+{\mathbf p} _2)\\ ({\mathbf q} _0+{\mathbf p} _2+{\mathbf p} _1)\otimes ({\mathbf q} _0+{\mathbf p} _2+{\mathbf p} _1)\\ \vdots \\ \left( {\mathbf q} _0+{\mathbf p} _2+(p^k-1){\mathbf p} _1\right) \otimes \left( {\mathbf q} _0+{\mathbf p} _2+(p^k-1){\mathbf p} _1\right) \\ \vdots \\ \left( {\mathbf q} _0+(p^k-1){\mathbf p} _2\right) \otimes \left( {\mathbf q} _0+(p^k-1){\mathbf p} _2\right) \\ \left( {\mathbf q} _0+(p^k-1){\mathbf p} _2+{\mathbf p} _1\right) \otimes \left( {\mathbf q} _0+(p^k-1){\mathbf p} _2+{\mathbf p} _1\right) \\ \vdots \\ \left( {\mathbf q} _0+(p^k-1){\mathbf p} _2+(p^k-1){\mathbf p} _1\right) \otimes \left( {\mathbf q} _0+(p^k-1){\mathbf p} _2+(p^k-1){\mathbf p} _1\right) \\ \vdots \\ \left( {\mathbf q} _0+(p^k-1){\mathbf p} _{2d}+\cdots +(p^k-1){\mathbf p} _1\right) \otimes \left( {\mathbf q} _0+(p^k-1){\mathbf p} _{2d}+\cdots +(p^k-1){\mathbf p} _1\right) \end{array}\right) \mod p^k.$$

After a succession of elementary row operations, namely left multiplication by the matrix below

11 $$\begin{aligned} P_1&= \left( \prod \limits _{u=2d-2}^1\prod \limits _{l=2d-1}^{u+1}\prod \limits _{i=2}^{p^{(2d-l)k}}E_{i+p^{(2d-u)k}+p^{(2d-l)k},p^{(2d-u)k}+p^{(2d-l)k}+1}(-1)\right) \\&\quad \times \left( \prod \limits _{u=2d-1}^1\left[ \prod \limits _{l=2d}^{u+1}\left( \prod \limits _{j=2}^{p^k-1}\prod \limits _{i=1}^{p^{(2d-l)k}}E_{p^{(2d-u)k}+jp^{(2d-l)k}+i,i+p^{(2d-u)k}+p^{(2d-l)k}}(-j)\right) \right. \right. \\ &\quad \times\;\;\left. \left. \left( \prod \limits _{j=1}^{p^k-1}\prod \limits _{i=1}^{p^{(2d-l)k}} E_{p^{(2d-u)k}+jp^{(2d-l)k}+i,i+p^{(2d-u)k}}(-1)\right) \right] \right) \\ &\quad \times \left( \prod \limits _{u=2d-1}^1\prod \limits _{j=2}^{p^k-1} \prod \limits _{i=2}^{p^{(2d-u)k}}E_{i+jp^{(2d-u)k},1+jp^{(2d-u)k}} (-1)\right) \\ &\quad \times \left( \prod \limits _{u=2d}^1 \left( \prod \limits _{j=2}^{p^k-1}\prod \limits _{i=1}^{p^{(2d-u)k}} E_{i+jp^{(2d-u)k},i+p^{(2d-u)k}}(-j)\right) \right. \\&\quad \times\;\;\left. \left( \prod \limits _{j=1}^{p^k-1}\prod \limits _{i=1}^{p^{(2d-u)k}} E_{i+jp^{(2d-u)k},i}(-1)\right) \right) , \end{aligned}$$

(10) can be reduced to the form $P_1\cdot {\mathcal {Q}}_{k,d}$, which only has the following nonzero rows

12 $$\begin{aligned} &{\mathbf q} _0 \otimes {\mathbf q} _0;\\& {\mathbf q} _0\otimes {\mathbf p} _i+{\mathbf p} _i\otimes {\mathbf q} _0+{\mathbf p} _i\otimes {\mathbf p} _i,\quad i=1,\ldots ,2d;\\ &{\mathbf p} _i\otimes {\mathbf p} _j+{\mathbf p} _j\otimes {\mathbf p} _i,\quad 2d\ge i>j\ge 1;\\ & (j^2-j){\mathbf p} _i\otimes {\mathbf p} _i,\quad i=1,\ldots ,2d,j=2,\ldots ,p^k-1.\\ \end{aligned}$$

From now we begin to investigate the solutions of the matrix equation (8) by distinguishing two cases: $p\ne 2$ and *p* = 2.

We first consider Case 1: $p\ne 2$.

By multiplying $P_1\cdot {\mathcal {Q}}_{k,d}$ on the left with the following matrix:

13 $$\begin{aligned} P_2&= \left( \prod \limits _{v=1}^{2d-2}\prod \limits _{u=0}^{v}\prod \limits _{i=p^{vk}+p^{uk}-(v+3)}^0E_{1+p^{vk}+p^{uk}+(v-u)-i+\sum \limits _{j=0}^{2d-2-v}(2d+1-j),p^{vk}+p^{uk}+(v-u)-i+\sum \limits _{j=0}^{2d-2-v}(2d+1-j)}\right) \\&\quad \times \left( \prod \limits _{u=0}^{2d-1}\prod \limits _{i=p^{(2d-1)k}+p^{uk}-(2d+2)}^0E_{1+p^{(2d-1)k}+p^{uk}+(2d-1-u)-i,p^{(2d-1)k}+p^{uk}+(2d-1-u)-i})\right) \\&\quad \times \left( \prod \limits _{u=1}^{2d-1}\prod \limits _{i=p^{uk}-2}^{0}E_{p^{uk}+2d-u-i,p^{uk}+2d-u-1-i}\right) \\&\quad \times \left( \prod \limits _{u=2d-1}^{0}\left[ (E_{1+p^{uk},1+2p^{uk}}(-1))\times \left( \prod \limits _{i=3}^{p^k-1}E_{1+ip^{uk},1+2p^{uk}}(i^2-i)\right) \right. \right. \\&\quad \left. \left. \times \left( E_{1+2p^{uk}}\left( \frac{p^k+1}{2}\right) \right) \right] \mod p^k\right) . \end{aligned}$$

$P_1\cdot {\mathcal {Q}}_{k,d}$ can be changed into the matrix $$\left( \begin{array}{c}\bar{\mathcal {Q}}_{k,d,p\ne 2}\\ {\mathbf{0}}_{(p^{2kd}-2d^2-3d-1)\times (4d^2+4d+1)}\end{array}\right)$$, where $\bar{\mathcal {Q}}_{k,d,p\ne 2}$ is of full row rank.

Write

$$P_2\cdot P_1\cdot [{\mathcal {Q}}_{k,d},b_1,\ldots ,b_d]=\left( \begin{array}{cccc}\bar{\mathcal {Q}}_{k,d,p\ne 2} &{} \bar{b}_1 &{} \cdots &{} \bar{b}_d\\ {\mathbf{0}} &{} \tilde{b}_1 &{}\cdots &{} \tilde{b}_d \end{array}\right) ,$$

then (8) has solution if and only if $[\tilde{b}_1, \ldots , \tilde{b}_d]={\mathbf{0}}_{(p^{2kd}-2d^2-3d-1)\times d}$.

Next, suppose (8) has solution, then the solution matrix can be obtained. Note that

$${\mathcal {Q}}_{k,d} [x_1,\ldots ,x_d]=[b_1,\ldots ,b_d]$$

is equivalent to the matrix equation

14 $$\bar{\mathcal {Q}}_{k,d,p\ne 2}[x_1,\ldots ,x_d]=[\bar{b}_1,\ldots ,\bar{b}_d].$$

Since the rank of $\bar{\mathcal {Q}}_{k,d,p\ne 2}$ is $2d^2+3d+1$, there exists an invertible matrix $Q_1$ of order $(2d+1)^2$, such that

15 $$\bar{\mathcal {Q}}_{k,d,p\ne 2}Q_1=\left[ I_{2d^2+3d+1},{\mathbf{0}}_{(2d^2+3d+1)\times (2d^2+d)}\right] ,$$

where

16 $$\begin{aligned} Q_1&= \left( \prod \limits _{j=0}^{2d-1}\prod \limits _{i=j+2}^{2d+1}E_{j(2d+1)+i,(i-1)(2d+1)+j+1}(-1)\right) \\&\quad \times \left( \prod \limits _{u=1}^{2d}\prod \limits _{j=u+1}^{2d+1}\prod \limits _{i=0}^{\left( \sum \nolimits _{l=1}^ul\right) - 1}E_{u(2d+1)+j-i,u(2d+1)+j-i-1}\right) . \end{aligned}$$

Obviously, (14) is equivalent to the matrix equation

17 $$\bar{\mathcal {Q}}_{k,d,p\ne 2}Q_1Q_1^{-1}[x_1,\ldots ,x_d]=[\bar{b}_1,\ldots ,\bar{b}_d].$$

Let $Q_1^{-1}[x_1,\ldots ,x_d]=[y_1,\ldots ,y_d]$, then (17) takes the form

18 $$\left[ I_{2d^2+3d+1},{\mathbf{0}}_{(2d^2+3d+1)\times (2d^2+d)}\right] [y_1,\ldots ,y_d]=[\bar{b}_1,\ldots ,\bar{b}_d].$$

According to the theory of linear system, the solution matrices of (18) can be represented by

19 $$[y_1,\ldots ,y_d]=\left( \begin{array}{cccc} \bar{b}_1&{} \bar{b}_2&{} \cdots &{} \bar{b}_d\\ k_{11}&{} k_{12}&{}\cdots &{}k_{1d}\\ k_{21} &{} k_{22} &{}\cdots &{}k_{2d}\\ \vdots &{}\vdots &{}\vdots &{}\vdots \\ k_{2d^2+d,1}&{}k_{2d^2+d,2}&{}\cdots &{} k_{2d^2+d,d} \end{array}\right) ,$$

where $k_{uv}$ are randomly selected from $GF(p^k)$, $(u=1,\ldots , 2d^2+d;v=1,\ldots ,d)$. Furthermore, (14) has the following solution matrices

20 $$[x_1,\ldots ,x_d]=Q_1\cdot \left( \begin{array}{cccc} \bar{b}_1&{} \bar{b}_2&{} \cdots &{} \bar{b}_d\\ k_{11}&{} k_{12}&{}\cdots &{}k_{1d}\\ k_{21} &{} k_{22} &{}\cdots &{}k_{2d}\\ \vdots &{}\vdots &{}\vdots &{}\vdots \\ k_{2d^2+d,1}&{}k_{2d^2+d,2}&{}\cdots &{} k_{2d^2+d,d} \end{array}\right) ,$$

namely,

21 $$[\overline{vec}({\mathcal {A}}_1),\ldots ,\overline{vec}({\mathcal {A}}_d)]=Q_1\cdot \left( \begin{array}{cccc} \bar{b}_1&{} \bar{b}_2&{} \cdots &{} \bar{b}_d\\ k_{11}&{} k_{12}&{}\cdots &{}k_{1d}\\ k_{21} &{} k_{22} &{}\cdots &{}k_{2d}\\ \vdots &{}\vdots &{}\vdots &{}\vdots \\ k_{2d^2+d,1}&{}k_{2d^2+d,2}&{}\cdots &{} k_{2d^2+d,d} \end{array}\right) .$$

Since $k_{uv}$ is sampled from $GF(p^k)$, $(u=1,\ldots , 2d^2+d;v=1,\ldots ,d)$, it is obvious that the number of such solution matrices is $p^{kd\cdot (2d^2+d)}$. For an arbitrary solution matrix, $\{{\mathcal {A}}_1,\ldots ,{\mathcal {A}}_d\}$ can be obtained immediately. Furthermore, by (4) we can obtain *d* quadratic functions of MQQ.

We summarize the above deduction for Case 1 as the following theorem.

#### **Theorem 2**

*Suppose* $$p\ne 2$$ *and*

$$P_2\cdot P_1\cdot [b_1,\ldots ,b_d]=\left( \begin{array}{ccc} \bar{b}_1 &{} \cdots &{} \bar{b}_d\\ \tilde{b}_1 &{}\cdots &{} \tilde{b}_d \end{array}\right) ,$$

*then* (8) *has solution if and only if* $[\tilde{b}_1 ,\ldots , \tilde{b}_d]={\mathbf{0}}_{(p^{2kd}-2d^2-3d-1)\times d}$ *. Furthermore, its solution are the matrices of the form*

22 $$[\overline{vec}({\mathcal {A}}_1),\ldots ,\overline{vec}({\mathcal {A}}_d)]=Q_1\cdot \left( \begin{array}{cccc} \bar{b}_1&{} \bar{b}_2&{} \cdots &{} \bar{b}_d\\ k_{11}&{} k_{12}&{}\cdots &{}k_{1d}\\ k_{21} &{} k_{22} &{}\cdots &{}k_{2d}\\ \vdots &{}\vdots &{}\vdots &{}\vdots \\ k_{2d^2+d,1}&{}k_{2d^2+d,2}&{}\cdots &{} k_{2d^2+d,d} \end{array}\right) ,$$

*where* $k_{uv}\in GF(p^k),(u=1,\ldots , 2d^2+d;v=1,\ldots ,d)$ *, and* $P_1,P_2,Q_1$ *are defined as* (11),(13) *and* (16).

Now we begin to consider Case 2: $p=2$.

By multiplying $P_1\cdot {\mathcal {Q}}_{k,d}$ on the left by the following matrix:

23 $$\begin{aligned} P_3&= \left( \prod \limits _{v=1}^{2d-2}\prod \limits _{u=0}^{v-1}\prod \limits _{i=p^{vk}+p^{uk}-(v+3)}^0E_{p^{vk}+p^{uk}+(v-u)-i+\sum \limits _{j=0}^{2d-2-v}(2d-j),p^{vk}+p^{uk}+(v-u)-i-1+\sum \limits _{j=0}^{2d-2-v}(2d-j)}\right) \\&\quad \times \left( \prod \limits _{u=0}^{2d-2}\prod \limits _{i=p^{(2d-1)k}+p^{uk}-(2d+2)}^0E_{p^{(2d-1)k}+p^{uk}+(2d-1-u)-i,p^{(2d-1)k}+p^{uk}+(2d-1-u)-i-1}\right) \\&\quad \times \left( \prod \limits _{u=1}^{2d-1}\prod \limits _{i=p^{uk}-2}^{0}E_{p^{uk}+2d-u-i,p^{uk}+2d-u-1-i}\right) \\&\quad \times \left( \prod \limits _{u=2d-1}^{0}\prod \limits _{i=2}^{p^k-1}E_{1+ip^{uk}}(p^{k-1})\mod p^k\right) , \end{aligned}$$

$P_1\cdot {\mathcal {Q}}_{k,d}$ can be changed into the matrix $$\left( \begin{array}{c}\bar{\mathcal {Q}}_{k,d,p=2}\\ {\mathbf{0}}_{(2^{2kd}-2d^2-d-1)\times (4d^2+4d+1)}\end{array}\right)$$, where $\bar{\mathcal {Q}}_{k,d,p=2}$ is of full row rank.

Write

$$P_3\cdot P_1\cdot [{\mathcal {Q}}_{k,d},b_1,\ldots ,b_d]=\left( \begin{array}{cccc}\bar{\mathcal {Q}}_{k,d,p=2} &{} \hat{b}_1 &{} \cdots &{} \hat{b}_d\\ {\mathbf{0}} &{} \check{b}_1 &{}\cdots &{} \check{b}_d \end{array}\right) ,$$

then (8) has solution if and only if $[\check{b}_1, \ldots , \check{b}_d]={\mathbf{0}}_{(2^{2kd}-2d^2-d-1)\times d}$.

Suppose (8) has solution, then we show how the solution matrix can be obtained. Since ${\mathcal {Q}}_{k,d} [x_1,\ldots ,x_d]=[b_1,\ldots ,b_d]$ is equivalent to the matrix equation

24 $$\bar{\mathcal {Q}}_{k,d,p=2}[x_1,\ldots ,x_d]=[\hat{b}_1,\ldots ,\hat{b}_d]$$

and the rank of $\bar{\mathcal {Q}}_{k,d,p=2}$ is $2d^2+d+1$, then there exists an invertible matrix $Q_2$ of order $(2d+1)^2$ such that

25 $$\bar{\mathcal {Q}}_{k,d,p=2}Q_2=\left[ I_{2d^2+d+1},{\mathbf{0}}_{(2d^2+d+1)\times (2d^2+3d)}\right] ,$$

where

26 $$\begin{aligned} Q_2&= \left( \prod \limits _{i=2}^{2d+1}E_{i,(i-1)(2d+1)+1}(-1)E_{i,(i-1)(2d+1)+i}(-1)\right) \\&\quad \times \left( \prod \limits _{j=1}^{2d-1}\prod \limits _{i=j+2}^{2d+1}E_{j(2d+1)+i,(i-1)(2d+1)+j+1}(-1)\right) \\&\quad \times \left( \prod \limits _{u=1}^{2d-1}\prod \limits _{j=u+2}^{2d+1}\prod \limits _{i=0}^{\left( \sum \limits _{l=2}^{u+1}l\right) -1}E_{u(2d+1)+j-i,u(2d+1)+j-i-1}\right) . \end{aligned}$$

Obviously, (24) is equivalent to the matrix equation

27 $$\bar{\mathcal {Q}}_{k,d,p=2}Q_2Q_2^{-1}[x_1,\ldots ,x_d]=[\hat{b}_1,\ldots ,\hat{b}_d].$$

Let $Q_2^{-1}[x_1,\ldots ,x_d]=[z_1,\ldots ,z_d]$, then (27) takes the form

28 $$\left[ I_{2d^2+d+1},{\mathbf{0}}_{(2d^2+d+1)\times (2d^2+3d)}\right] [z_1,\ldots ,z_d]=[\hat{b}_1,\ldots ,\hat{b}_d].$$

According to the theory of linear system, the solution matrices of (28) can be represented by

29 $$[z_1,\ldots ,z_d]=\left( \begin{array}{cccc} \hat{b}_1&{} \hat{b}_2&{} \cdots &{} \hat{b}_d\\ k_{11}&{} k_{12}&{}\cdots &{}k_{1d}\\ k_{21} &{} k_{22} &{}\cdots &{}k_{2d}\\ \vdots &{}\vdots &{}\vdots &{}\vdots \\ k_{2d^2+3d,1}&{}k_{2d^2+3d,2}&{}\cdots &{} k_{2d^2+3d,d} \end{array}\right) ,$$

where $k_{uv}$ is sampled from $GF(2^k)$ $(u=1,\ldots , 2d^2+3d;v=1,\ldots ,d)$. Furthermore, (24) has the following solution matrices

30 $$[x_1,\ldots ,x_d]=Q_2\cdot \left( \begin{array}{cccc} \hat{b}_1&{} \hat{b}_2&{} \cdots &{} \hat{b}_d\\ k_{11}&{} k_{12}&{}\cdots &{}k_{1d}\\ k_{21} &{} k_{22} &{}\cdots &{}k_{2d}\\ \vdots &{}\vdots &{}\vdots &{}\vdots \\ k_{2d^2+3d,1}&{}k_{2d^2+3d,2}&{}\cdots &{} k_{2d^2+3d,d} \end{array}\right) ,$$

namely,

31 $$[\overline{vec}({\mathcal {A}}_1),\ldots ,\overline{vec}({\mathcal {A}}_d)]=Q_2\cdot \left( \begin{array}{cccc} \hat{b}_1&{} \hat{b}_2&{} \cdots &{} \hat{b}_d\\ k_{11}&{} k_{12}&{}\cdots &{}k_{1d}\\ k_{21} &{} k_{22} &{}\cdots &{}k_{2d}\\ \vdots &{}\vdots &{}\vdots &{}\vdots \\ k_{2d^2+3d,1}&{}k_{2d^2+3d,2}&{}\cdots &{} k_{2d^2+3d,d} \end{array}\right) .$$

Since $k_{uv}$ is sampled from $GF(2^k)$, $(u=1,\ldots , 2d^2+3d;v=1,\ldots ,d)$, it is obvious that the number of such solution matrices is $2^{kd\cdot (2d^2+3d)}$. For an arbitrary solution matrix, $\{{\mathcal {A}}_1,\ldots ,{\mathcal {A}}_d\}$ can be got immediately. Furthermore, according to (4) we can obtain *d* quadratic functions of MQQ.

We summarize the above deduction for Case 2 as the following theorem.

#### **Theorem 3**

*Suppose* $$p=2$$ *and*

$$P_3\cdot P_1\cdot [b_1,\ldots ,b_d]=\left( \begin{array}{ccc} \hat{b}_1 &{} \cdots &{} \hat{b}_d\\ \check{b}_1 &{}\cdots &{} \check{b}_d \end{array}\right) ,$$

*then* (8) *has solution if and only if* $[\check{b}_1 ,\ldots , \check{b}_d]={\mathbf{0}}_{(2^{2kd}-2d^2-d-1)\times d}$ *. Furthermore, its solution are the matrices of the form*

32 $$[\overline{vec}({\mathcal {A}}_1),\ldots ,\overline{vec}({\mathcal {A}}_d)]=Q_2\cdot \left( \begin{array}{cccc} \hat{b}_1&{} \hat{b}_2&{} \cdots &{} \hat{b}_d\\ k_{11}&{} k_{12}&{}\cdots &{}k_{1d}\\ k_{21} &{} k_{22} &{}\cdots &{}k_{2d}\\ \vdots &{}\vdots &{}\vdots &{}\vdots \\ k_{2d^2+3d,1}&{}k_{2d^2+3d,2}&{}\cdots &{} k_{2d^2+3d,d} \end{array}\right) ,$$

*where* $k_{uv}\in GF(2^k),(u=1,\ldots , 2d^2+3d;v=1,\ldots ,d)$ *, and* $P_1,P_3,Q_2$ *are defined as* (11),(23) *and* (26).

To end this section, we summarize our proposed algorithm as follows:

#### **Algorithm 1**

Algorithm for checking whether a given quasigroup of order $GF(p^{kd})$ is an MQQ over $GF(p^{k})$

1. Write the given quasigroup of order *p* ^*kd*^ in a vector with the form of (3).
2. Convert every element of the vector into a *d*-ary sequence over $GF(p^{k})$, then a $p^{2kd}\times d$ Boolean matrix $[b_1,\ldots ,b_d]$ is obtained, where every $b_s(s=1,\ldots , d)$ is $p^{2kd}$ dimensional column vector.
3. If $p\ne 2$, for given *k* and *d*, compute the corresponding $P_1,P_2,Q_1$ according to (11),(13) and (16).
4. Compute $$P_2\cdot P_1\cdot [b_1,\ldots ,b_d]=\left( \begin{array}{ccc} \bar{b}_1 &{} \cdots &{} \bar{b}_d\\ \tilde{b}_1 &{}\cdots &{} \tilde{b}_d \end{array}\right)$$.
5. If $[\tilde{b}_1 ,\ldots , \tilde{b}_d]\ne {\mathbf{0}}_{(p^{2kd}-2d^2-3d-1)\times d}$, then output “*no MQQ*”.
6. If $[\tilde{b}_1 ,\ldots , \tilde{b}_d]={\mathbf{0}}_{(p^{2kd}-2d^2-3d-1)\times d}$, choose randomly $k_{uv}\in GF(p^k),(u=1,\ldots , 2d^2+d;v=1,\ldots ,d)$, and compute $$Q_1\cdot \left( \begin{array}{cccc} \bar{b}_1&{} \bar{b}_2&{} \cdots &{} \bar{b}_d\\ k_{11}&{} k_{12}&{}\cdots &{}k_{1d}\\ k_{21} &{} k_{22} &{}\cdots &{}k_{2d}\\ \vdots &{}\vdots &{}\vdots &{}\vdots \\ k_{2d^2+d,1}&{}k_{2d^2+d,2}&{}\cdots &{} k_{2d^2+d,d} \end{array}\right) =[\overline{vec}({\mathcal {A}}_1),\ldots ,\overline{vec}({\mathcal {A}}_d)]$$.
7. Write out $\{{\mathcal {A}}_1,\ldots ,{\mathcal {A}}_d\}$ according to $[\overline{vec}({\mathcal {A}}_1),\ldots ,\overline{vec}({\mathcal {A}}_d)]$.
8. Compute $\{f_1,\ldots ,f_d\}$ by (4) and output “$f_1,\ldots ,f_d$ *of MQQ*”.
9. If $p=2$, compute $P_1,P_3,Q_2$ according to (11), (23) and (26).
10. Compute $$P_3\cdot P_1\cdot [b_1,\ldots ,b_d]=\left( \begin{array}{ccc} \hat{b}_1 &{} \cdots &{} \hat{b}_d\\ \check{b}_1 &{}\cdots &{} \check{b}_d \end{array}\right)$$.
11. If $[\check{b}_1 ,\ldots , \check{b}_d]\ne {\mathbf{0}}_{(2^{2kd}-2d^2-d-1)\times d}$, then output “*no MQQ*” .
12. If $[\check{b}_1 ,\ldots , \check{b}_d]={\mathbf{0}}_{(2^{2kd}-2d^2-d-1)\times d}$, choose randomly $k_{uv}\in GF(2^k),(u=1,\ldots , 2d^2+3d;v=1,\ldots ,d)$, and compute $$Q_2\cdot \left( \begin{array}{cccc} \hat{b}_1&{} \hat{b}_2&{} \cdots &{} \hat{b}_d\\ k_{11}&{} k_{12}&{}\cdots &{}k_{1d}\\ k_{21} &{} k_{22} &{}\cdots &{}k_{2d}\\ \vdots &{}\vdots &{}\vdots &{}\vdots \\ k_{2d^2+3d,1}&{}k_{2d^2+3d,2}&{}\cdots &{} k_{2d^2+3d,d} \end{array}\right) =[\overline{vec}({\mathcal {A}}_1),\ldots ,\overline{vec}({\mathcal {A}}_d)]$$.
13. Write out $\{{\mathcal {A}}_1,\ldots ,{\mathcal {A}}_d\}$ according to $[\overline{vec}({\mathcal {A}}_1),\ldots ,\overline{vec}({\mathcal {A}}_d)]$.
14. Compute $\{f_1,\ldots ,f_d\}$ by (4) and output “$f_1,\ldots ,f_d$ *of MQQ*”.

## Two examples

In this section, we use two examples which are dealing with quasigroups of order $2^4$ and $3^2$ respectively, to illustrate the validity of the theorems and the effectiveness of the proposed algorithm.

### Example 1

A quasigroup $(Q,*)$ of order $2^4$ and its corresponding representations based on $GF(2^2)$ are given in Table 2.

**Table 2:** A quasigroup $(Q,*)$ of order 2^4^ and its representations based on $GF(2^2)$

*	0	1	2	3	4	5	6	7	8	9	10	11	12	13	14	15
0	0	1	2	3	4	5	6	7	8	9	10	11	12	13	14	15
1	1	0	3	2	5	4	7	6	9	8	11	10	13	12	15	14
2	2	3	0	1	6	7	4	5	10	11	8	9	14	15	12	13
3	3	2	1	0	7	6	5	4	11	10	9	8	15	14	13	12
4	4	5	6	7	0	1	2	3	12	13	14	15	8	9	10	11
5	5	4	7	6	1	0	3	2	13	12	15	14	9	8	11	10
6	6	7	4	5	2	3	0	1	14	15	12	13	10	11	8	9
7	7	6	5	4	3	2	1	0	15	14	13	12	11	10	9	8
8	8	9	10	11	12	13	14	15	0	1	2	3	4	5	6	7
9	9	8	11	10	13	12	15	14	1	0	3	2	5	4	7	6
10	10	11	8	9	14	15	12	13	2	3	0	1	6	7	4	5
11	11	10	9	8	15	14	13	12	3	2	1	0	7	6	5	4
12	12	13	14	15	8	9	10	11	4	5	6	7	0	1	2	3
13	13	12	15	14	9	8	11	10	5	4	7	6	1	0	3	2
14	14	15	12	13	10	11	8	9	6	7	4	5	2	3	0	1
15	15	14	13	12	11	10	9	8	7	6	5	4	3	2	1	0

*	00	01	02	03	10	11	12	13	20	21	22	23	30	31	32	33
00	00	01	02	03	10	11	12	13	20	21	22	23	30	31	32	33
01	01	00	03	02	11	10	13	12	21	20	23	22	31	30	33	32
02	02	03	00	01	12	13	10	11	22	23	20	21	32	33	30	31
03	03	02	01	00	13	12	11	10	23	22	21	20	33	32	31	30
10	10	11	12	13	00	1	02	03	30	31	32	33	20	21	22	23
11	11	10	13	12	01	0	03	02	31	30	33	32	21	20	23	22
12	12	13	10	11	02	03	00	01	32	33	30	31	22	23	20	21
13	13	12	11	10	03	02	01	00	33	32	31	30	23	22	21	20
20	20	21	22	23	30	31	32	33	00	01	02	03	10	11	12	13
21	21	20	23	22	31	30	33	32	01	00	03	02	11	10	13	12
22	22	23	20	21	32	33	30	31	02	03	00	01	12	13	10	11
23	23	22	21	20	33	32	31	30	03	02	01	00	13	12	11	10
30	30	31	32	33	20	21	22	23	10	11	12	13	00	01	02	03
31	31	30	33	32	21	20	23	22	11	10	13	12	01	00	03	02
32	32	33	30	31	22	23	20	21	12	13	10	11	02	03	00	01
33	33	32	31	30	23	22	21	20	13	12	11	10	03	02	01	00

Suppose $$P_3\cdot P_1\cdot [b_1,b_2]=\left( \begin{array}{cc} \hat{b}_1 &{} \hat{b}_2\\ \check{b}_1 &{} \check{b}_2 \end{array}\right) .$$ Since $(\check{b}_1 , \check{b}_2)={\mathbf{0}}_{245,2}$, according to Theorem 3, the quasigroup is a MQQ. For a random matrix

$$(k_{uv})_{14\times 2}=\left( \begin{array}{cccccccccccccc} 3&{}1&{}2&{}1&{}3&{}2&{}1&{}0&{}2&{}1&{}2&{}2&{}3&{}2\\ 1&{}1&{}3&{}3&{}1&{}3&{}0&{}1&{}2&{}0&{}0&{}1&{}1&{}2\\ \end{array}\right) ^T,$$

the corresponding functions are achieved as follows:

$$\begin{aligned} f_1&= (1,x_1,x_2,x_3,x_4){\mathcal {A}}_1\left( \begin{array}{c}1\\ x_1\\ x_2\\ x_3\\ x_4\end{array}\right) =(1,x_1,x_2,x_3,x_4)\left( \begin{array}{ccccc} 0 &{} 1 &{} 3 &{}1 &{}1\\ 3&{} 1 &{} 3&{}1&{}2\\ 2 &{}1 &{} 3&{}0&{}2\\ 2&{}1&{}0&{}2&{}1\\ 1&{}2&{}2&{}3&{}2 \end{array}\right) \left( \begin{array}{c}1\\ x_1\\ x_2\\ x_3\\ x_4\end{array}\right) \\&= x_2+3x_3+2x_4+x_1^2+3x_2^2+2x_1x_3+2x_3^2+2x_4^2,\\ f_2&= (1,x_1,x_2,x_3x_4){\mathcal {A}}_1\left( \begin{array}{c}1\\ x_1\\ x_2\\ x_3\\ x_4\end{array}\right) =(1,x_1,x_2,x_3x_4)\left( \begin{array}{ccccc} 0 &{} 2 &{} 1 &{}3 &{}3\\ 1&{} 1 &{} 1 &{}0 &{}0\\ 3 &{}3 &{} 1 &{}3 &{}1\\ 3&{}0&{}1&{}2&{}3\\ 0&{}0&{}1&{}1&{}2 \end{array}\right) \left( \begin{array}{c}1\\ x_1\\ x_2\\ x_3\\ x_4\end{array}\right) \\&= 3x_1+2x_3+3x_4+x_1^2+x_2^2+2x_2x_4+2x_3^2+2x_4^2. \end{aligned}$$

### Example 2

A quasigroup $(Q,*)$ of order $3^2$ and its corresponding representations based on *GF*(3) are given in Table 3.

**Table 3:** A quasigroup $(Q,*)$ of order $3^2$ and its representations based on *GF*(3)

*	0	1	2	3	4	5	6	7	8
0	0	1	2	3	4	5	6	7	8
1	1	2	0	4	5	3	7	8	6
2	2	0	1	5	3	4	8	6	7
3	3	4	5	6	7	8	0	1	2
4	4	5	3	7	8	6	1	2	0
5	5	3	4	8	6	7	2	0	1
6	6	7	8	0	1	2	3	4	5
7	7	8	6	1	2	0	4	5	3
8	8	6	7	2	0	1	5	3	4

*	00	01	02	10	11	12	20	21	22
00	00	01	02	10	11	12	20	21	22
01	01	02	00	11	12	10	21	22	20
02	02	00	01	12	10	11	22	20	21
10	10	11	12	20	21	22	00	01	02
11	11	12	10	21	22	20	01	02	00
12	12	10	11	22	20	21	02	00	01
20	20	21	22	00	01	02	10	11	12
21	21	22	20	01	02	00	11	12	10
22	22	20	21	02	00	01	12	10	11

Suppose $$P_2\cdot P_1\cdot [b_1,b_2]=\left( \begin{array}{cc} \hat{b}_1 &{} \hat{b}_2\\ \check{b}_1 &{} \check{b}_2 \end{array}\right) .$$ Since $(\check{b}_1 , \check{b}_2)={\mathbf{0}}_{66,2}$, according to Theorem 2, the quasigroup is an MQQ. For a random matrix $(k_{uv})_{10\times 2}\in GF(3)$

$$(k_{uv})_{10\times 2}=\left( \begin{array}{cccccccccc} 2&{}0&{}1&{}1&{}2&{}2&{}1&{}0&{}2&{}1\\ 1&{}2&{}2&{}1&{}0&{}1&{}2&{}2&{}1&{}2\\ \end{array}\right) ^T,$$

the corresponding functions are achieved as follows:

$$\begin{aligned} f_1&= (1,x_1,x_2,x_3,x_4){\mathcal {A}}_1\left( \begin{array}{c}1\\ x_1\\ x_2\\ x_3\\ x_4\end{array}\right) =(1,x_1,x_2,x_3,x_4)\left( \begin{array}{ccccc} 0 &{} 2 &{} 0 &{}0 &{}2\\ 2&{} 0 &{} 2&{}1 &{}0\\ 0 &{}1 &{}0 &{}1 &{}1\\ 1&{}2&{}2&{}0&{}2\\ 1&{}0&{}2&{}1&{}0 \end{array}\right) \left( \begin{array}{c}1\\ x_1\\ x_2\\ x_3\\ x_4\end{array}\right) \\&=x_1+x_3,\\ f_2&= (1,x_1,x_2,x_3,x_4){\mathcal {A}}_1\left( \begin{array}{c}1\\ x_1\\ x_2\\ x_3\\ x_4\end{array}\right) =(1,x_1,x_2,x_3,x_4)\left( \begin{array}{ccccc} 0 &{} 2 &{} 2 &{}2 &{}2\\ 1&{} 0 &{} 1 &{}0 &{}1\\ 2 &{}2 &{} 0 &{}2 &{}2\\ 1&{}0&{}1&{}0&{}1\\ 2&{}2&{}1&{}2&{}0 \end{array}\right) \left( \begin{array}{c}1\\ x_1\\ x_2\\ x_3\\ x_4\end{array}\right) \\&=x_2+x_4. \end{aligned}$$

## Conclusion

In this paper, a necessary and sufficient condition, which reveals that a given quasigroup $(Q,*)$ of order *p* ^*kd*^ is an MQQ over $GF(p^k)$ of type $Quad_{d-k}Lin_k$ if and only if the matrix equation (8) has solution, has been established. This condition provides a deep insight into the relationship between MQQ and the corresponding multiplication table from the point of view of quasigroup theory. Based on this condition, an algorithm has been developed to justify whether the given quasigroup is an MQQ over $GF(p^{k})$ and generate the polynomials if it is. Compared with the previous work (Zhang and Zhang 2013), this algorithm can identify both bilinear MQQs and non-bilinear ones, and the algebraic operation in Galois fields provides more flexibility in choosing *p*, *k* and *d*, which is beneficial for applying MQQ-design to various platforms. The validity of the theorems and the effectiveness of the proposed algorithm have been verified by two examples.
